# Case Report: A rare chromosomal imbalance with dup 7q36.3-qter and del 7pter-p22.3 arising from parental pericentric inversion

**DOI:** 10.3389/fgene.2025.1564711

**Published:** 2025-07-17

**Authors:** Rongbo Lin, Wenhui Zhang, Mingwei Huang, Yansheng Shen, Jianxiang Liao, Ping Song, Ying Qi, Jie He, Yuanxiang Xia, Jing Duan, Yuanzhen Ye, Qiuwei Yi, Pei Lan, Lingyu Kong, Zhanqi Hu

**Affiliations:** ^1^ Department of Neurology, Shenzhen Children’s Hospital, Shenzhen, China; ^2^ Department of Emergency, Shenzhen Children’s Hospital, Shenzhen, China; ^3^ Department of Pediatrics, Shenzhen Guangming District People’s Hospital, Shenzhen, China; ^4^ Aegicare (Shenzhen) Technology Co., Ltd., Shenzhen, China; ^5^ Department of Respiratory Medicine, Shenzhen Children’s Hospital, Shenzhen, China

**Keywords:** neurodevelopmental delay, facial dysmorphism, chromosome 7 imbalance, dup 7q36.3-qter, del 7pter-p22.3, parental pericentric inversion, genotype-phenotype correlation, genetic counseling

## Abstract

Chromosomal abnormality is a significant cause of neurodevelopmental delay and congenital malformation. Only a few cases of chromosome 7 imbalances with both duplication of the distal long arm (7q) and deletion of the distal short arm (7p) have been reported without a systematic analysis of the genotype-phenotype relationship. We identify a new case of chromosome 7 imbalance with dup 7q36.3-qter and del 7pter-p22.3 and thoroughly characterize the chromosomal abnormality in the patient and related family members using a variety of genetic tests. More importantly, similar cases of 7q duplication and 7p deletion arising from parental pericentric inversion are reviewed to clarify the genotype-phenotype correlation of the disease. In summary, in cases of normal prenatal and early postnatal growth, progressive neurodevelopmental delay, intellectual disability, limited speech, and mild facial dysmorphism, the rare combination of duplication and deletion of distal ends of chromosome 7 may be suspected. Parental pericentric chromosomal inversion is likely a genetic contributor to the duplication-deletion imbalance in the offspring despite normal phenotypes in the inversion carrier, so genetic testing and counseling are recommended for better disease management and prevention.

## 1 Introduction

Only a few cases of chromosome 7 imbalances with both duplication of the distal long arm (7q) and deletion of the distal short arm (7p) have been reported previously, thus making the diagnosis and disease management of those patients and future ones challenging. The first two documented patients in 1978 were from a single big family and carried the exact duplication of 7q32-qter and deletion of 7pter-p22. By traditional chromosomal banding and meiotic analysis, the abnormalities in chromosome 7 were revealed as a result of meiotic crossing-over and recombination of a parental pericentric inversion, inv(7)(p22q32) (Winsor et al., 1978). Subsequent occasional reports on different cases of chromosome 7 duplication-deletion described similar distal short-arm deletion of 7pter-p22 but varying lengths of long-arm duplication (from 7q11.22-qter to 7q35-qter), which possibly explain the broad spectrum of clinical phenotypes (Ishii et al., 1997; Goodman et al., 1999; Lukusa et al., 2002; Tchirikov et al., 2010). The most recent case study identified a stillborn boy with a very large duplication that included almost the entire long arm of chromosome 7, who showed severe prenatal growth retardation, micrognathia, ventricular septal defect, aortic coarctation, bradyarrhythmia, pericardial effusion, bilateral hydronephrosis, infravesical obstruction, and cerebellar hypoplasia (Tchirikov et al., 2010). To date, no systematic analysis has been conducted on those patients to clarify the potential genotype-phenotype relationship.

In this study, we report on a new case of chromosome 7 imbalance with a small duplication of distal 7q36.3 and deletion of distal 7p22.3. Notable clinical features of our proband include progressive neurodevelopmental delay, mild facial dysmorphism, and drug-controlled seizures. A variety of genetic tests are carried out to thoroughly characterize the chromosomal abnormality in the patient and related family members. We also review similar cases of 7q duplication and 7p deletion arising from parental pericentric inversion to clarify the genotype-phenotype correlation of the disease.

## 2 Materials and methods

### 2.1 Whole genome sequencing (WGS) and whole exome sequencing (WES)

Genomic DNA was isolated from peripheral blood samples using a previously published method (Duan et al., 2022). WGS and WES were performed on the BGI DNBSEQ-T7RS sequencer platform on MGIEasy universal DNA library for the proband and his parents, respectively.

### 2.2 The SNP array

The CNVs were detected by Affymetrix CytoScan750K_Array (Affymetrix, Santa Clara, CA). The SNP array was performed on DNA isolated from the proband’s EDTA-treated blood (Zhang et al., 2021). Genomic DNA was extracted using the DNeasy Blood & Tissue Kit (Qiagen, GmbH, Germany), then quantified, digested, ligated, fragmented, labeled, hybridized, stained, and scanned following the Affymetrix protocol.

### 2.3 The optical genome mapping (OGM)

Fresh peripheral blood from the patient was collected and stored. Subsequently, ultra-high molecular weight DNA was isolated, labeled, uploaded, and scanned. Labeled DNA was especially uploaded to nanochannel chips and scanned using a Saphyr instrument (Bionano Genomics). Finally, image analysis was performed using the Bionano *de novo* genome assembly pipeline. The genome maps obtained were aligned and assembled with Human Genome Reference Consortium GRCh37/hg19 for structural variants (SVs) detection.

### 2.4 The translocation breakpoints analysis

Genomic DNA was isolated from peripheral blood from the patient and two healthy boys as controls, purified, amplified, and quantified. Subsequently, the polymerase chain reaction (PCR) products were displayed with 2% agarose gel electrophoresis and analyzed using Sanger sequencing. The PCR and Sanger sequencing primers, spanning the translocation breakpoints, were CGG​CGG​CTT​CCC​CTC​CTT​CC (forward) and GCT​GCC​GCT​GTC​CCT​CCA​CC (reverse).

## 3 Result

### 3.1 Clinical presentation

**FIGURE 1:**
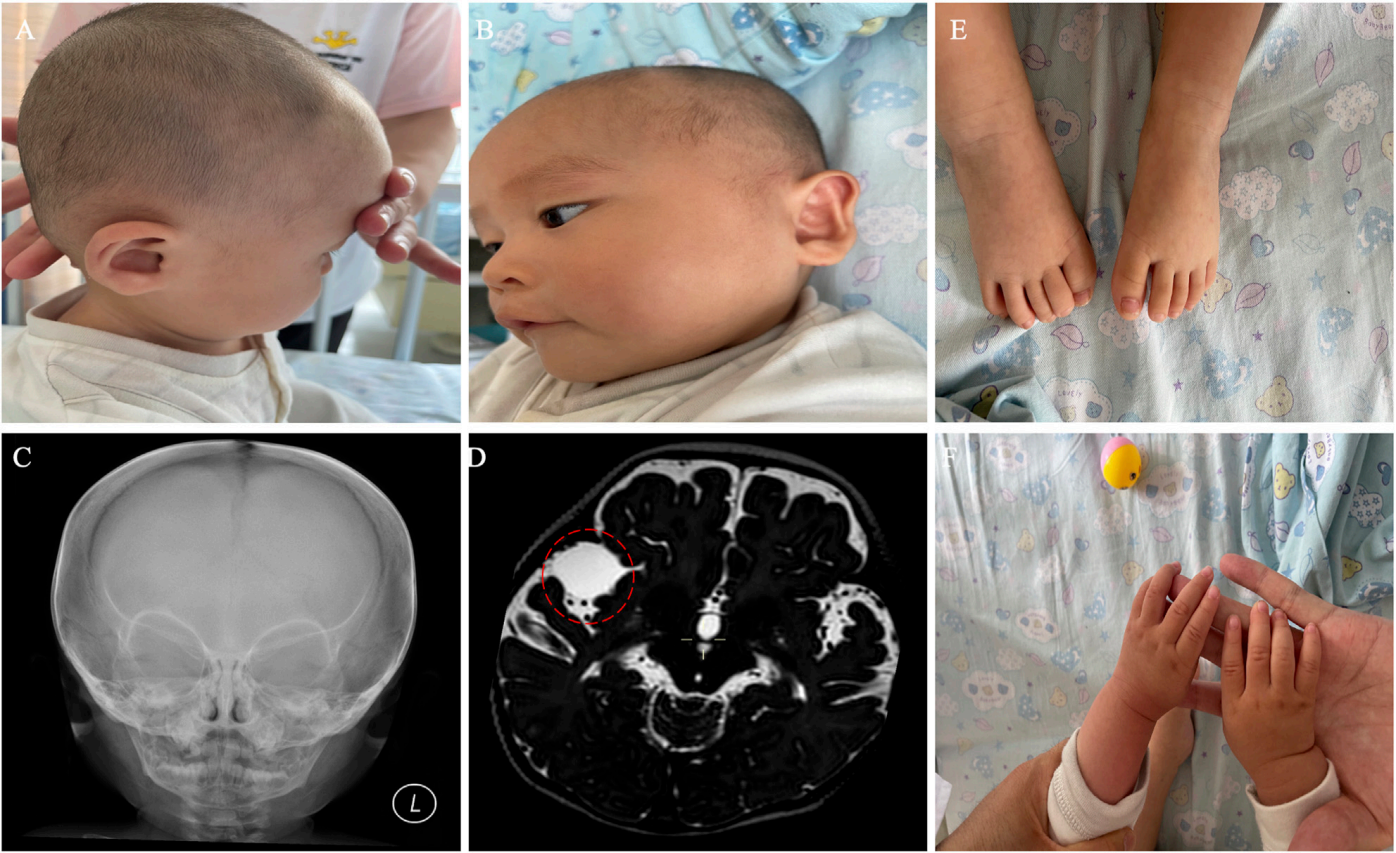
Clinical phenotypes of the proband with 7p22.3 deletion and 7q36.3 duplication at 10 months of age. **(A,B)** Dysmorphic facial features of the proband. **(C)** X-ray of the face. **(D)** Brain MRI showed a middle cranial fossa meningioma. The dashed red circle marks the approximate boundaries of the region associated with a middle cranial fossa meningioma. **(E,F)** normal trunk and limbs.

We present the case of a 3-year-old boy with no notable family history of disease. He was born full-term via cesarean section to a healthy mother. Typical developmental milestones were achieved before the age of 5 months. However, following the first episode of seizure, progressive neurodevelopmental delay was observed, characterized by intellectual disability, limited speech, distinctive facial features, and a middle cranial fossa meningioma (Figure 1). His facial dysmorphism included a high and prominent forehead, large fontanels with wide cranial sutures, a round face, a high-arched palate, bitemporal narrowing, an arched mouth, a short and prominent nose, and small, low-set ears (Figures 1A–C). No abnormalities were detected in the trunk or limbs (Figures 1E,F). Systematic laboratory tests ruled out ocular or congenital heart diseases. His seizures have been successfully controlled with sodium valproate oral solution (Sanofi, 3.5 mL orally, twice daily).

### 3.2 Chromosomal abnormalities determined through multiple tests

**FIGURE 2:**
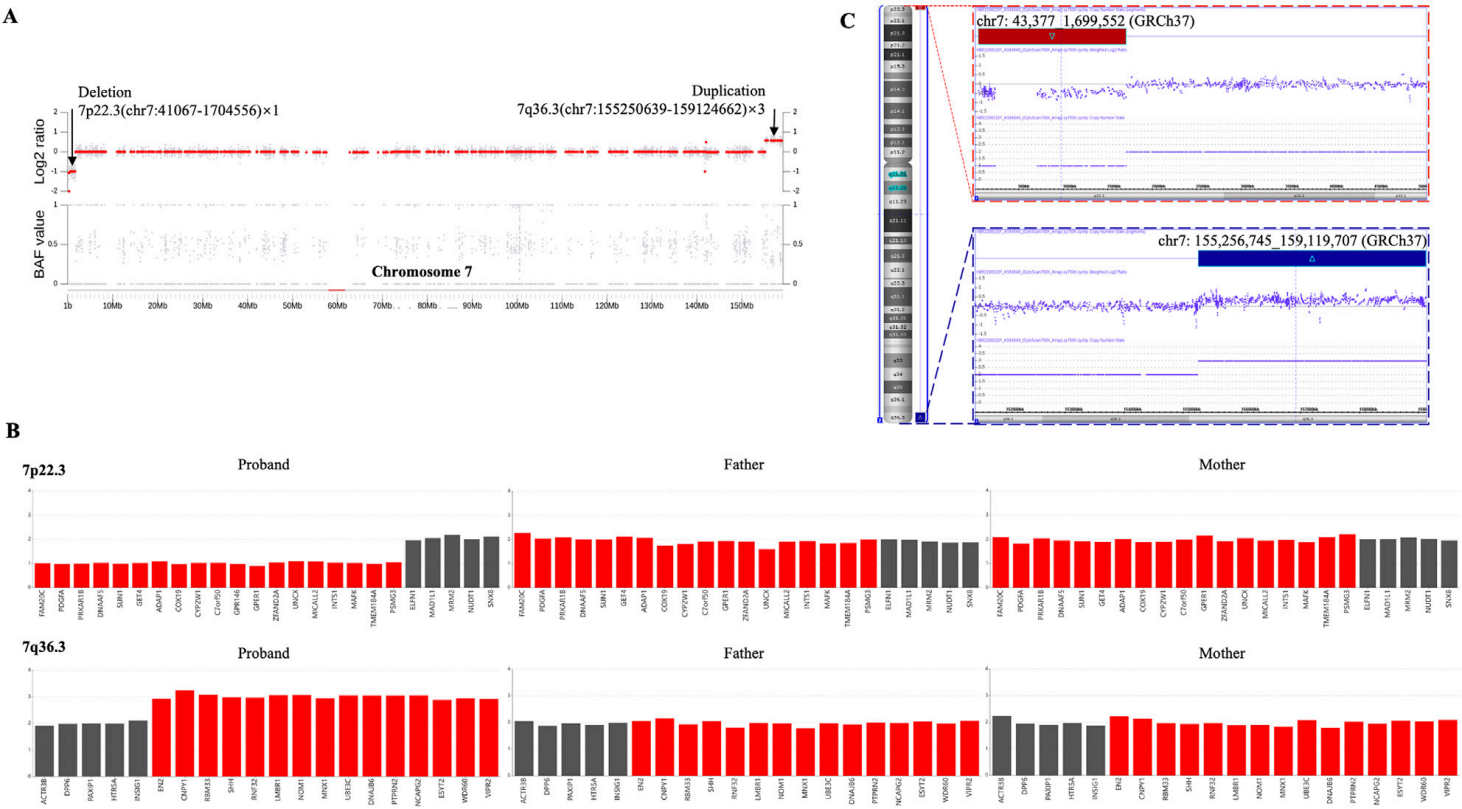
The heterozygous 7p22.3 deletion and 7q36.3 duplication were identified via CNV analysis utilizing next-generation sequencing (NGS) and SNP array in a male patient. **(A)** Scatter diagram of the CNV analysis with NGS data. This diagram shows the log2 ratio of sample/batch median (upper) and BAF value (lower) for chromosome 7, where deleted 7p22.3 and duplicated 7q36.3 are marked with chromosome coordinates (GRCh37). **(B)** The CNV variants from the proband are absent in both of his parents. This diagram shows the copy numbers for genes within deleted 7p22.3 (upper) and duplicated 7q36.3 (lower). Individual genes within each diagram are arranged according to their sequential locations on the chromosome. Red bars indicate genes affected by the CNV deletion/duplication in the patient, whereas black bars indicate genes unaffected. **(C)** CNVs were confirmed by SNP array. This diagram shows the log2 ratio of sample/batch median for chromosome 7, where deleted 7p22.3 (red) and duplicated 7q36.3 (blue) are marked with chromosome coordinates (GRCh37).

To identify potential genetic causes of the disease, WGS and WES were performed on the index patient and his unaffected parents, respectively, with a mean sequencing depth of 35% and 98.98% of target coding regions being sequenced 15 times or more. No single nucleotide variants or small insertions/deletions were found to match the clinical presentations described above. However, we identified two copy number variants (CNVs) in the patient, a 7p22.3 deletion (1.66 Mb) and a 7q36.3 duplication (3.87 Mb), both of which were absent in his parents (Figures 2A,B). Subsequently, both CNVs were further confirmed by single nucleotide polymorphism (SNP) array analysis using CytoScan 750K Array (Figure 2C). Due to the relatively small sizes of our identified CNVs, we did not carry out traditional karyotyping such as GTG banding, which typically has a low resolution (5–10 Mb) (Mantere et al., 2021).

**FIGURE 3:**
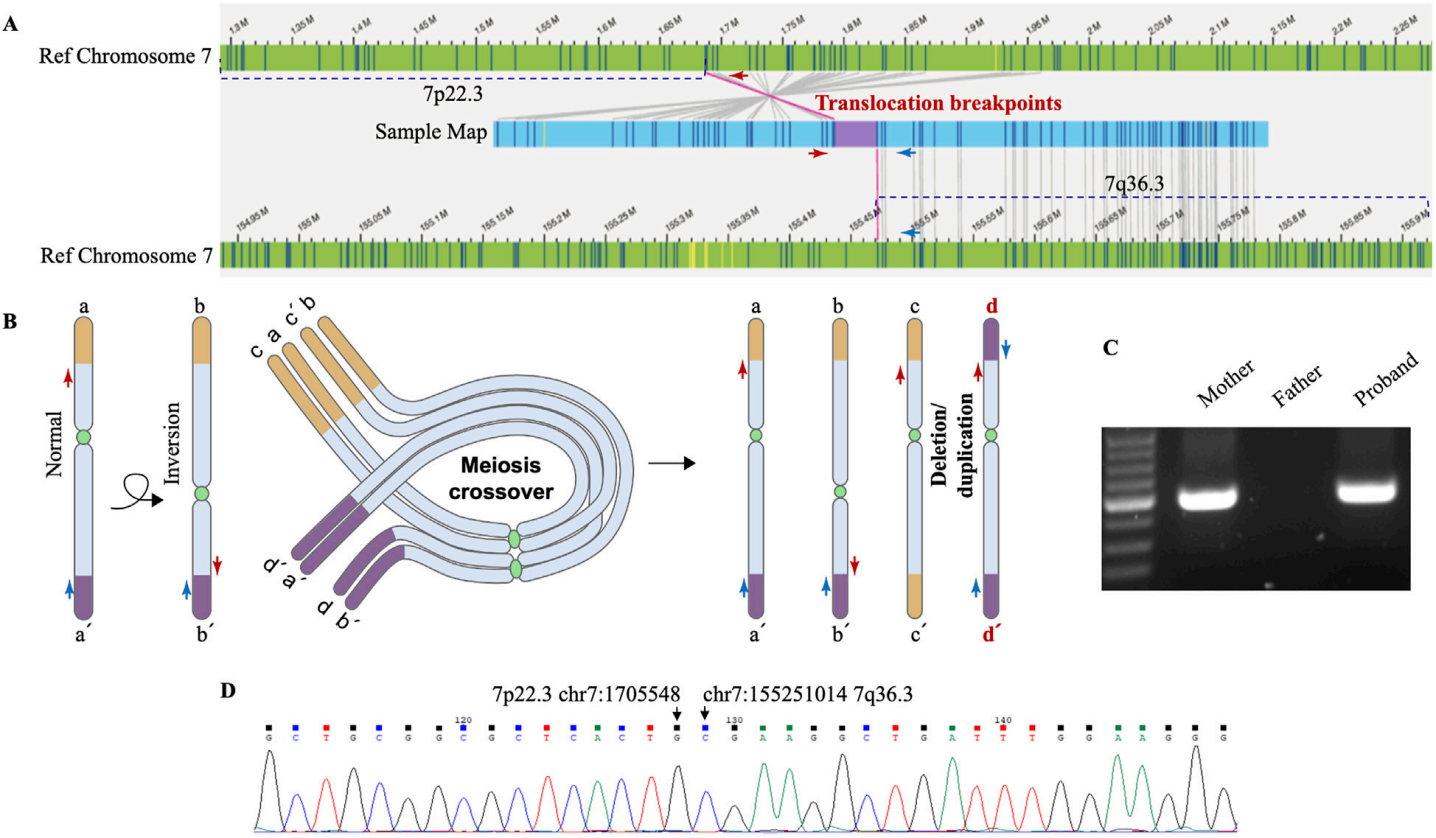
Genomic rearrangements disclosed by OGM and verified by Sanger sequencing. **(A)** OGM detects an intrachromosomal fusion of two distal chromosome 7 fragments (Sample map, blue ribbon), which matches a region next to the deleted 7p22.3 (left) and the duplicated 7q36.3 (right). The upper reference OGM map of normal Chromosome 7 (green ribbons) shows the deleted 7p22.3 (marked with a dashed line) and the neighboring region, and the lower reference map (green ribbons) shows the duplicated 7q36.3 (marked with a dashed line) and the neighboring region. Bands within the blue and green ribbons indicate specific labeling patterns of different chromosome regions via OGM. The matching of bands (gray connecting lines) suggests identical sequence compositions between compared regions. OGM does not specify the exact translocation breakpoints within the purple region. PCR primers designed to span the assumed translocation breakpoints are shown as red and blue arrows at corresponding chromosome regions. **(B)** Schematic representation of a proposed mechanism for how deletion/duplication imbalances of Chromosome 7 in the proband result from a balanced chromosome inversion carried by a parent. Normal chromosome (a-a’) may undergo pericentric inversion to form a balanced product (b-b’). In a heterozygous carrier, a-a’ and b-b’ can pair up in a loop configuration during meiosis, when a single crossover may lead to the production of four gametes, including a-a’ and b-b’, as well as recombinant c-c’ and d-d’ with deletion/duplication imbalances. The distal ends of the short and long arms of chromosome 7 are marked as yellow and purple, respectively. d-d’ with deleted 7p22.3 and duplicated 7q36.3 is present in the proband. Red and blue arrows indicate PCR primers for breakpoint verification and sequencing. **(C)** PCR results verify chromosome 7 translocation in the proband and inversion in his mother. PCR amplification was carried out using primers indicated above and DNA isolated from the proband’s and his parents’ blood samples. The results were analyzed by 2% agarose gel electrophoresis, and the size of the expected amplification product is 291 bp. **(D)** The exact breakpoints are identified by Sanger sequencing. DNA was gel purified from the bands shown above and then subjected to Sanger sequencing. The breakpoints in hg19 are chr7: (G)1705548 (p22.3) and chr7: (G)155251014 (q36.3), as indicated by arrows.

Despite the identification of both CNVs, the localization and orientation of the duplicated 7q36.3 region could not be determined by WGS or SNP array. Therefore, optical genome mapping (OGM), which images very long linear DNA libraries (median size larger than 250 kb) and allows comprehensive identification of structural variants (Mantere et al., 2021), was performed on the patient blood sample. As illustrated in Figure 3A, OGM identified an intrachromosomal fusion of two distal chromosome 7 fragments, which matches the duplicated 7q36.3 and a region next to the deleted 7p22.3 (fus(7; 7)(p22.3; q36.3)), suggesting the duplicated 7q36.3 is translocated to where the deleted 7p22.3 normally locates.

The translocation breakpoints are located within a region marked as purple inside the sample map of the fused fragment (Figure 3A), but OGM does not reveal a nucleotide-resolution sequence of the region due to its technological limitation. As such, PCR primers were designed to span the breakpoints so that Sanger sequencing of the PCR products could reveal the precise breakpoints. Besides, previous cases of intrachromosomal deletion-duplication imbalances of chromosome 7 all originated from a parent with chromosome 7 pericentric inversion. We proposed a mechanism for how deletion/duplication imbalances of Chromosome 7 in the proband results from a balanced chromosome inversion carried by a parent (Figure 3B). PCR results verified chromosome 7 translocation in the proband and inversion in his mother rather than his father (Figure 3C), supporting maternal balanced chromosomal changes as a source of chromosome imbalances in the proband. More importantly, Sanger sequencing of the PCR products identified the exact sequence of the breakpoints (Figure 3D), which are chr7: g.1705548 (p22.3) and chr7: g.155251014 (q36.3).

## 4 Discussion

To the best of our knowledge, our patient is the only documented case of chromosome 7 imbalances with distal 7p22.3 deletion and 7q36.3 duplication as a result of parental chromosome pericentric inversion and subsequent meiotic crossing-over and recombination. Table 1 compiles similar cases of chromosome 7 imbalances with both 7p deletion and 7q duplication, including those previously reported in the literature and our proband. To illustrate the potential genotype-phenotype relationship, these cases are arranged according to the size of the unbalanced regions, from large to small.

**TABLE 1:** Cases of chromosome 7 imbalances with both 7p deletion and 7q duplication.

References	Proband karyotype	Clinical presentations
Tchirikov et al. (2010)	rec(7)dup(7q)inv(7)(p22.3q11.22)mat by traditional chromosomal banding, fluorescence *in situ* hybridization (FISH), and array comparative genomic hybridization (CGH)	Severe prenatal growth retardation, micrognathia, ventricular septal defect, aortic coarctation, bradyarrhythmia, pericardial effusion, bilateral hydronephrosis, infravesical obstruction, cerebellar hypoplasia, disproportion with a large head, and oligohydramnios during pregnancy; stillbirth at 38 weeks of gestation
Ishii et al. (1997)	rec(7)dup(7q)inv(7)(p22q22)pat by traditional chromosomal banding	Hydramnios during pregnancy; Severe growth retardation, hypotonia, and respiratory distress at birth at 40 weeks of gestation; abnormal facial features, microretrognathia, short neck, severely contracted limbs, atrial septal defect, scoliosis and chondrodysplasia punctata of the carpal, femoral, and vertebrosacral bones; died at 1 month of age due to hypoventilation
Goodman et al. (1999)	rec(7)dup(7q)inv(7)(p22q31.3)pat by traditional chromosomal banding and FISH	Fetal spine abnormality, ambiguous genitalia, and dilation of the lateral cerebral ventricles and the cisterna magna by prenatal ultrasound; at birth after 37 weeks of gestation, macrocephaly with a large anterior fontanelle, facial dysmorphism, microretrognathia, short neck, limb abnormalities, dilated lateral cerebral ventricles and cisterna magna, bilateral optic nerve atrophy and retinal degeneration, a small secundum atrial septal defect, and bilateral nephrocalcinosis; Postnatal growth retardation, facial dysmorphism, swallowing dysfunction, hypotonia, hypogonadotrophic hypogonadism, gastroesophageal reflux and chronic pulmonary disease (last evaluated at 8 months of age)
Winsor et al. (1978)	rec(7)dup(7q)inv(7)(p22q32)pat (rec(7)dup(7q)inv(7)(p22q32)mat for her female first cousin) by traditional chromosomal banding	Apparently normal at birth, feeding difficulty, slow weight gain; severe subnormal growth, facial dysmorphism, scoliosis, rib abnormalities, hypotonic limbs with increased mobility of joints and valgus deformity of the feet at 3 years of age A 23-year old cousin, severely handicapped and subnormal, small stature, similar facial dysmorphism (high and arched palate, prognathism) but no scoliosis or rib abnormalities
Lukusa et al. (2002)	rec(7)dup(7q)inv(7)(p22q35) by traditional chromosomal banding and FISH	Normal prenatal growth; kyphosis, developmental/mental retardation, and abnormal ears (last evaluated at 29 years of age)
Present study	rec(7)dup(7q)inv(7)(p22.3q36.3)mat by next-generation sequencing and OGM	Normal prenatal and postnatal growth until 5 months of age, progressive neurodevelopmental delay, intellectual disability, limited speech, distinctive facial features, and a middle cranial fossa meningioma

It is interesting to note that all surveyed cases present with distal 7p22 deletion despite the fact that the patients are unrelated to each other. Since the deletion-duplication imbalances presumably arise from parental chromosome pericentric inversion, which has been confirmed in nearly all parents, we postulate that 7p22 may be a hotspot for chromosome inversion. Our study identifies the exact sequence of the breakpoints with the help of OGM and Sanger sequencing. In contrast, previous reports only utilized more traditional chromosomal banding and/or complementary methods to map the approximate locations (Table 1). Applying high-precision tools such as OGM in future cases will enable better characterization and verification of the 7p22 inversion breakpoints.

Because the compiled cases have similar distal 7p22 deletions, their different clinical presentations could mainly be attributed to differences in distal 7q duplications. Despite interpersonal variability and various genetic backgrounds, a marked proportionality between the size of 7q duplication and the survival and prenatal/postnatal growth is observed, consistent with an earlier study on patients with partial trisomy 7q (Forabosco et al., 1988). The most severe case with the largest 7q duplication, 7q11.22-qter, that included almost the entire long arm of chromosome 7, showed dramatic prenatal growth retardation, cerebellar hypoplasia, micrognathia, aortic coarctation, ventricular septal defect, hydronephrosis, and stillbirth at 38 weeks of gestation (Tchirikov et al., 2010). Slightly shorter duplication of 7q22-qter correlated with severe growth retardation at birth, abnormal facial features, microretrognathia, short neck, severely contracted limbs, atrial septal defect, scoliosis, and premature death at 1 month of age (Ishii et al., 1997). A case with decreased duplication of 7q31.3-qter presented with significant prenatal and postnatal growth retardation, facial dysmorphism, and abnormalities in multiple body systems but survived to at least 8 months of age by the last evaluation (Goodman et al., 1999). With even shorter duplication of 7q (7q32-qter and 7q35-qter), the patients were apparently normal at birth, showed seemingly fewer clinical symptoms, and survived to older ages (Winsor et al., 1978; Lukusa et al., 2002). In our case of 7q36.3 duplication, the shortest one identified so far, normal prenatal and postnatal growth was observed until 5 months of age, when progressive neurodevelopment delay and intellectual disability started to manifest. Even though the proband is only 3 years of age currently, his relatively mild symptoms suggest that he could survive to an old age. Follow-ups of the cases compiled in Table 1 and future investigation of new cases will significantly enrich the genetic and phenotypic spectrums of chromosome 7 imbalances with both 7p deletion and 7q duplication, promoting a better understanding of the genotype-phenotype correlation of the disease.

Of the two breakpoints identified in our study, chr7: g.1705548 (p22.3) maps to 145 bp upstream of the *ELFN1* gene, whereas chr7: g.155251014 (q36.3) maps to the first exon of *EN2*. Considering the mother’s overall healthy condition, we reason that these breakpoints in a heterozygous state do not lead to detrimental disruption in gene regulation or expression. Similarly, other parental carriers of balanced chromosome 7 pericentric inversions presented with no notable clinical symptoms (Winsor et al., 1978; Ishii et al., 1997; Goodman et al., 1999; Lukusa et al., 2002; Tchirikov et al., 2010). However, under extremely rare circumstances, homozygosity in pericentric inversion of chromosome 7 may lead to significant disruption in a *HOXA13* enhancer sequence and contribute to disease in a patient with hand-foot-genital syndrome (Watson et al., 2016). More importantly, such pericentric inversions carry well-known risks for duplication and/or deletion of chromosome fragments in the offspring through crossing-over and recombination during meiosis (Liehr et al., 2019; Ishii et al., 1997; Goodman et al., 1999). Therefore, mapping out the inversion breakpoints as precisely as possible in the carriers would be of critical value for genetic counseling and disease prevention. Even though the telomeric repeat sequences can render such nuanced genomic characterization challenging in our case, an integrated approach with complementary tools such as NGS, OGM, SNP array, and PCR-based Sanger sequencing would meet the need.

Notable genes within the 1.66 Mb CNV deletion of 7p22.3 include *FAM20C*, *PDGFA*, *PRKAR1B*, *DNAAF5*, *SUN1*, *GET4*, *ADAP1*, *COX19*, *CYP2W1*, *C7orf50*, *GPR146*, *GPER1*, *ZFAND2A*, *UNCX*, *MICALL2*, *INTS1*, *MAFK*, *TMEM184A*, and *PSMG3* (Figure 2B), but none of them is haploinsufficient individually according to the Clinical Genome Resource (ClinGen) (Wright et al., 2024). Chromosome 7p22.3 deletions alone have been associated with neurodevelopmental delays and cardiac defects (Skvortsova et al., 2024; Mastromoro et al., 2020). More specifically, a minimal deleted region of less than 200 kb, which spans *MAD1L1*, *FTSJ2*, *NUDT1*, and *SNX8*, was delineated for cardiac anomalies (Richards et al., 2011), but a later case study on a smaller deletion involving the *SNX8* gene supported *SNX8* haploinsufficiency in neurodevelopment rather than cardiac development (Mastromoro et al., 2020). In our case of neurodevelopmental delay without apparent cardiac defects, the region from *MAD1L1* to *SNX8* was unaffected by the deletion (Figure 2B). In contrast, a recent case of 7p22.3 deletion, which includes this region from *MAD1L1* to *SNX8*, reported both neurodevelopmental delay and heart anomalies (Skvortsova et al., 2024), supporting the likely involvement of the *MAD1L1*-*SNX8* region in cardiac development (but cannot exclude the possible role of *SNX8* in neurodevelopment). Among the genes affected by our 7p22.3 deletion, *PRKAR1B* is of particular interest due to its established autosomal dominant inheritance pattern in Marbach-Schaaf neurodevelopmental syndrome (Marbach et al., 2021). It is suggested that *PRKAR1B* haploinsufficiency is the primary mechanism underlying the intellectual disability phenotype for 7p22.3 deletion (Skvortsova et al., 2024). However, the identification of only missense variants for Marbach-Schaaf neurodevelopmental syndrome and the abundant presence of loss-of-function variants (nonsense, frameshift, and splice variants) in the population database gnomAD (https://gnomad.broadinstitute.org/gene/ENSG00000188191) indicate that *PRKAR1B* is not haploinsufficient, consistent with its low statistical values for haploinsufficiency index and probability of loss-of-function intolerance (https://search.clinicalgenome.org/kb/genes/HGNC:9390). Other genes with an established autosomal recessive inheritance pattern, such as *FAM20C* and *INTS1*, could not be the primary cause of disease in our case of 7p22.3 deletion either.

Similarly, the 3.87 Mb CNV duplication of 7q36.3 include *EN2*, *CNPY1*, *RBM33*, *SHH*, *RNF32*, *LMBR1*, *NOM1*, *MNX1*, *UBE3C*, *DNAJB6*, *PTPRN2*, *NCAPG2*, *ESYT2*, *WDR60*, and *VIPR2* (Figure 2B), but there is no sufficient evidence for triplosensitivity for any of the individual genes by ClinGen (Wright et al., 2024). Therefore, we reason that no deletion or duplication of single genes is responsible for the disease presentation in our case, where the disturbed expression and regulation of multiple genes ultimately lead to disease. With this reasoning, our case of chromosome 7 abnormalities could be syndromic, because several body systems would be affected by this broad range of genetic disruptions, even though only minor symptoms have manifested at the current age. Future enrichment of the genetic and phenotypic spectrums of related cases, together with better molecular tools to define the chromosomal abnormalities, may contribute to identifying candidate genes for genotype-phenotype correlation.

## 5 Conclusion

In summary, in cases of normal prenatal and early postnatal growth, progressive neurodevelopmental delay, intellectual disability, limited speech, and mild facial dysmorphism, the rare combination of duplication and deletion of distal ends of chromosome 7 may be suspected. Parental pericentric chromosomal inversion is likely a genetic contributor to the duplication-deletion imbalance in the offspring despite normal phenotypes in the inversion carrier, so genetic testing and counseling are recommended for better disease management and prevention.

## Data Availability

The data presented in the study are deposited in the Genome Sequence Archive in National Genomics Data Center, China National Center for Bioinformation / Beijing Institute of Genomics, Chinese Academy of Sciences that are publicly accessible at https://bigd.big.ac.cn/gsa-human/browse/HRA012222.
